# Pokémon GO Within the Context of Family Health: Retrospective Study

**DOI:** 10.2196/10679

**Published:** 2018-10-03

**Authors:** Lisa K Militello, Nathan Hanna, Claudio R Nigg

**Affiliations:** 1 Martha S Pitzer Center for Women, Children, and Youth College of Nursing The Ohio State University Columbus, OH United States; 2 College of Nursing The Ohio State University Columbus, OH United States; 3 Office of Public Health Studies University of Hawaii Honolulu, HI United States

**Keywords:** family, pediatrics, mHealth, exercise, mobile health, public health

## Abstract

**Background:**

Pokémon GO illuminated the potential for mobile phone gaming apps to engage users and promote health. However, much work is needed to fully understand the mechanisms through which digitally supported behavior change interventions operate, particularly for children and families.

**Objective:**

The aims of this study were (1) to explore the Pokémon GO user experience from a family perspective and (2) to investigate Pokémon GO within the context of family health.

**Methods:**

Between January and February 2017, congruent with one of the largest anticipated Pokémon GO updates Gen 2, participants were recruited from parks, word of mouth, and social media to complete a Web-based survey. Participants were surveyed about family characteristics, interest, and experiences playing Pokémon GO and healthy lifestyle beliefs. Using a revised Godin Leisure-Time Exercise Questionnaire, a retrospective pre-post design assessed changes in parent physical activity (PA) before and after playing Pokémon GO.

**Results:**

Self-reported data from 160 parents and 31 children were included in the final analyses (representing 129 parents and 31 parent-child dyads). Gameplay most often occurred between sons aged 10 years or younger and mothers. “Spending time together” was the most cited reason for gameplay by both parents (122/160, 76.3%) and children (24/31, 77%), followed by “it helped me go outdoors” for parents (113/160, 70.1%) and “I am a Pokémon fan” by children (21/31, 68%). Interestingly, open-ended responses indicated that gameplay could trigger both positive and negative emotional parent response. The most cited reason for app disengagement was boredom; conversely, the most cited reason for app re-engagement was in-app events. For parents, there were significant increases in minutes spent in mild (mean 23.36 [SD 66.02]; t_97_=3.50, *P*<.001) and moderate (mean 21.76 [SD 53.04]; t_130_=4.70, *P*<.001) PA per week after playing Pokémon GO. However, child perceptions of parental influence on PA most significantly associated with parents who reported weekly strenuous PA both before (r_s_=.514, *P*=.003) and after (r_s_=.536, *P*=.003) Pokémon GO uptake.

**Conclusions:**

Pokémon GO transcended traditional understanding of digital health and uniquely reached across generations to engage users. Findings from this study highlight that, for a period of time, Pokémon GO fostered social and physical well-being for children and families through a multifaceted approach.

## Introduction

### Background

Health and behavior are interrelated. Globally, unhealthy lifestyle choices such as poor diet and physical inactivity are associated with chronic disease and reduced mortality [1]. In families, parent and child health are also interrelated through shared genetic and environmental factors [2-4]. There is strong evidence that parenting self-efficacy (belief in one’s ability) and parenting behavior are key social mechanisms in the intergenerational transmission of self-regulation and consistently correlate with a wide range of parent and child physical and psychological outcomes [5-7]. Stemming from decades of empirical support and grounded in theoretical foundations [2,8,9], core behavior change strategies frequently used to promote healthy lifestyle choices in both parents and children include, but are not limited to, self-monitoring, goal setting, social support, and the promotion of self-regulation and self-efficacy [2,10-13]. Given the shift toward precision medicine and ubiquitous nature of mobile phones, there is growing interest in the utility of mobile phones to monitor, assess, and support delivery of core behavior change strategies [14-16].

Most Americans (95%) are *connected* via mobile phones and other mobile devices, and more than half (62%) of the mobile phone owners have used their phone to get health information [17]. Mobile phone and Web-based media consume a significant portion of leisure time for individuals, couples, and families [18], with increasingly more families connecting *on the go* [19]. Nearly all children below the age of 8 years live in a home with some type of mobile device and on an average spend approximately 48 min per day on mobile devices each day [20]. Due to advances in technology, our understanding of human behavior is more dynamic than static to account for the ever-changing biological, social, personal, and contextual states associated with human behavior [21]. However, much work is needed to fully understand the mechanisms through which digitally supported behavior change interventions influence behavior in everyday real-world settings [22,23]. In particular, the design of many behavior intervention technologies focuses on individual health and does not address family health in a unified manner [3]. Yet, technology and online media provide a context for people to jointly create meaningful connections [23].

Stemming from the large multimedia Pokémon franchise, Pokémon GO became a global sensation in 2016 with estimates of 32 to 65 million monthly players at the peak of its popularity [24,25]. In Pokémon GO, players search for game-related characters or animations that have been overlaid onto real-world images (ie, augmented reality) via global positioning system capabilities of a mobile device [24,26,27]. Game play that requires physical activity (PA) is also referred to as active video games and can be useful in promoting PA when played as designed [24,28,29]. Not promoted as a *health* app, there is evidence to support significant changes in PA before and after playing Pokémon GO, as well as improvements in psychological health and cognitive performance [30-40]. As such, Pokémon GO sparked several conversations, hypotheses, and theories regarding motivation to play and the potential for similar games to synergize behavior change interventions [24,41-45].

A number of Pokémon GO studies have been conducted in college-aged students and young adults [32,36,37,39,40,46,47]. Fewer studies have examined Pokémon GO gameplay in pediatric populations [26,48,49] or within the context of family health. From the limited Pokémon GO family research, it is evident that gameplay “wasn’t really about the Pokémon.” [26]. In both parent and child Pokémon GO players, there is empirical support for socialization and family bonding associated with gameplay [26,48]. Pokémon GO also provided families with opportunities for exercise and outdoor time [26,48]. Although parental concerns related to injury prevention were commonly reported (eg, accidents and stranger danger) [26,48], concerns were often reconciled as family gameplay was perceived to be *different* than other types of screen time, promoting exercise, cooperation, and enjoyable exploration [26,48].

Pokémon GO succeeded in providing both spontaneous and planned opportunities across settings for parents and children to use media to create, discover, and mutually engage [26]. Engagement is critical to the success of any digitally supported behavior change intervention. Engagement may be conceptualized as the intervention itself (content and delivery), the context (setting and population in which the intervention is used), and the targeted behavior [50]. Joint media engagement (JME) refers to people using media together through a variety of spontaneous and designed experiences [23]. A working assumption of JME is that what goes on between people and around media can be as important as the content designed into the media [23]. If Pokémon GO gameplay for families was motivated by factors outside of the game itself, it is valuable to explore factors and context that led to initial engagement, trends over time, and associated health benefits.

### Objectives

Using family as a locus for JME and building upon previous literature, we investigated the Pokémon GO user experience from a family perspective and within the context of family health. We extended the science by exploring both parent and child perspectives of gameplay, investigated plausible theories of behavior change, and learned more about the potential for behavior change technologies that engage both the child and parent to support family health. Recommendations for future behavior change technologies aimed at promoting family health are presented.

## Methods

### Study Design

Descriptive survey data were collected from parents or adult caregivers “parents” who were impacted by a child or teen “child” that played Pokémon GO. The parent survey was used to understand family characteristics, parent experiences with Pokémon GO, and parent beliefs toward engaging in healthy lifestyle behaviors with their child. In addition, retrospective pre-post design was used to investigate changes in parent PA before and after playing Pokémon GO. Children and teens “children” who played Pokémon GO were also invited to participate. The child survey was used to understand child experiences with Pokémon GO as well as their perceptions of parental influence on PA. Surveys were disseminated to participants via a Qualtrics survey link. Methods and Results are described according to the Checklist for Reporting Results of Internet E-Surveys [51].

### Ethics

The Ohio State University Institutional Review Board approved this study. Before participation, electronic consent was obtained from parents. Child assent and parental consent were required for participation of the child. Children could complete the survey with the assistance of a parent or research team member (semistructured interview). All participants opted to complete the consents, assents, and surveys electronically and remotely (vs in-person).

### Subjects

One of the largest updates to Pokémon GO, Gen 2, was projected to release in February 2017. In an effort to coalesce recruitment and data collection with viral trends associated with Pokémon GO, an 8-week recruitment occurred between January and February 2017. Participants were recruited from the research team–initiated efforts to include word of mouth, social media announcements, and email blasts. In addition, fliers were disseminated among local parks, libraries, and coffee shops in urban (eg, Columbus, OH) and suburban neighborhoods (eg, suburban Ann Arbor, MI). Parents were included if they were 18 years old or older, spoke or read English, and had a child who played Pokémon GO. Children were included if they were 5 to 17 years old, played Pokémon GO (currently or in the past), and spoke or read English. Children were not required to have a parent in the study to participate. However, all children enrolled had a parent participate. Parents could earn US $20 and children could earn US $10 in gift cards for their participation.

### Data Collection and Measurements

Participants were asked to complete a one-time data collection via a survey link. Participants were able to toggle forward and back to review or change answers. Internet protocol (IP) addresses were used to reduce the likelihood of duplicate entry from the same user. Similar to other key protected health information identifiers (eg, phone number and email address), IP addresses were removed from the dataset and not used to identify any individuals.

Parents were asked to complete 3 surveys consisting of both open- and close-ended questions:

- *Family’s characteristics and Pokémon GO user experience* were obtained from a survey developed from techniques used in anthropology and human-computer interaction. The initial popularity and widespread penetration of Pokémon GO provided opportunities to observe users in the wild. The principal investigator engaged in need finding and *deep hanging out* [52-54] and created a Pokémon GO account, using the app with her child, spending time in parks and neighborhoods. To learn more about Pokémon GO itself, information was gathered from general press releases related to gameplay; visits to the Niantic Pokémon GO webpage; and trends, blogs, and feedback on social media. Iterative assessments of survey questions were discussed within a small academic Pokémon GO collaborative, which included colleagues similarly interested in exploring the Pokémon GO phenomenon for scientific value. A family snapshot was obtained from general demographic questions and questions related to family interests or goals. We inquired about the Pokémon GO user experience from the parent perspective (parent as a player), “Do you periodically check Pokémon GO throughout the day?” and parent perceptions of their child’s gameplay, “Does your child play Pokémon GO on his/her own phone or parent/adult phone?”
- *Healthy lifestyle beliefs scale* [55] was used to assess parental beliefs toward their ability to engage in healthy lifestyle behaviors (eg, “I believe I can help my child to lead a healthy life.” “I believe that I can reach the health goals that I set for myself.”). Participants responded to each item on a Likert scale ranging from 1 (strongly disagree) to 5 (strongly agree). Composite scores were averaged and presented as means, SDs, with higher scores indicative of stronger beliefs. Cronbach alphas have consistently been above .80 [56].
- A revised version of the *Godin-Shepard Leisure-Time Physical Activity Questionnaire* [57] was used to assess retrospective pre-post design and to investigate changes in parent PA since playing Pokémon GO. Days per week (0 to 7) and minutes per day (in 10-min intervals from 0 to 60+) spent in strenuous, moderate, and mild PA before and after beginning to play Pokémon GO were self-reported. A Leisure Score Index (LSI) was determined by multiplying the frequency of each level of PA by a corresponding metabolic equivalent of task value: 3 (mild), 5 (moderate), and 9 (strenuous) [27]. Individuals with LSI greater than or equal to 24 are recognized as active, whereas individuals with LSI less than or equal to 23 are classified as insufficiently active [58]. Three additional questions assessed hours of daily sedentary behavior before playing Pokémon GO [38].

Children were asked to complete 2 surveys consisting of both open- and close-ended questions:

- *Child characteristics and Pokémon GO user experience* were assessed via 19 questions consisting of demographic questions and questions related to experiences playing Pokémon GO. For example, children were asked to indicate, “Who do you typically play Pokémon GO with?” Similarly, “Did you participate in any Pokémon GO holiday events (eg, Pokémon GO Halloween?)? Please respond yes or no.” An open-ended question appeared at the end of the survey, “Is there anything else you want to share with us about why you play Pokémon GO?”
- *Parental influence on physical activity scale* (14 questions; Cronbach alpha >.70) [59,60] captures parental influence on a child’s PA via 4 subscales: general parenting support, active parents, past activity, and guiding support. Composite scores were averaged for a mean score, with higher scores indicative of greater support.

### Analysis

Data were analyzed using descriptive analyses, including means, SDs, and frequencies. Spearman rank correlation (rho) was used to assess relationships among study variables. Qualitative data were limited, stemming from open-ended survey questions. When possible, descriptive analysis of survey comments was used to identify recurrent themes and to highlight anecdotal feedback. As missing data were minimal and to ensure study quality, only data that were 90% or more completed were included in the final analysis, yielding a 98% parent and 94% child completion rate.

## Results

### Family Characteristics

Self-reported data from 160 parents and 31 children were included in the final analyses (representing 129 parents and 31 parent-child dyads). Both parent (141/160, 88.1%) and child (27/31, 87%) samples were overwhelmingly white. Over 60% of household incomes earned greater than US $50,000 annually, with half of that reporting incomes greater than US $100,000, and nearly 35% of the parents had a 4-year college degree. Tables 1-3 provide the family characteristics.

**Table 1:** Self-reported parent characteristics (n=160).

Characteristics		n (%)
**Age (years)**		
	18-24	14 (8.8)
	25-34	65 (40.6)
	35-44	58 (36.2)
	>45	23 (14.4)
**Gender**		
	Female	115 (71.9)
	Male	45 (28.1)
**Race or ethnicity**		
	Black	5 (3.1)
	White	141 (88.1)
	Hispanic	4 (2.5)
	Asian	3 (1.9)
	Native American	3 (1.9)
	Other	3 (1.9)
	Missing	1 (0.6)

**Table 2:** Parent reporting on their child’s characteristics (n=160).

Characteristics		n (%)
**Age (years)**		
	≤10	107 (66.8)
	11-15	35 (21.9)
	≥16	11 (6.9)
	Missing	7 (4.4)
**Gender**		
	Female	46 (28.8)
	Male	109 (68.1)
	Missing	5 (3.1)
**School level by grade**		
	Elementary (up to fifth)	123 (76.8)
	Middle (sixth to eighth)	20 (12.5)
	High school (ninth to twelfth)	14 (8.8)
	Other	3 (1.9)

**Table 3:** Self-reported child characteristics (n=31).

Characteristics^a^		n (%)
**Age, in years**		
	≤10	22 (71)
	11-15	6 (19)
	≥16	3 (10)
**Gender**		
	Female	7 (23)
	Male	24 (77)
**School level by grade**		
	Elementary (up to fifth)	22 (71)
	Middle (sixth to eighth)	4 (13)
	High school (ninth to twelfth)	5 (16)
**Race or ethnicity**		
	White	27 (87)
	Black	1 (3)
	Hispanic or Spanish	1 (3)
	Asian	2 (7)

^a^Data for children consented and assented into the study.

### User Experiences

For aim 1, data reflect facets of user experiences (eg, adoptability, accessibility, desirability, usability, and value) [54,61]. Before Pokémon GO, the majority of parents (142/156, 91.0%) and children (24/31, 77%) never played an augmented reality game. Parents indicated that more than half of the children borrowed a parent’s phone for gameplay (82/154, 53.3%), whereas 46.7% (72/154) of children played on their own phone. The majority of parents (79/155, 51.0%) downloaded the game within a week of release, whereas the majority of children downloaded the game within a month of release (12/30, 40%). However, nearly a fifth of both parents (28/155, 18.1%) and children (6/30, 20%) downloaded the game on the day it was released. Over 40% (69/160, 43.1%) of parents never engaged with the Pokémon brand before Pokémon GO. Conversely, children identified with the Pokémon brand through television shows (28/31, 90%), card collecting (23/31, 74%), and/or books (16/31, 52%). Table 4 highlights aspects of gameplay in families.

Open-ended questions provided insight into motivation and rationale for disengagement and re-engagement with the game (Table 5). Unique to family gameplay, parenting behaviors such as screen-time monitoring or leveraging the game for child reward or punishment (eg, “child punishment was over”) influenced engagement. Parent comments suggested that boredom was the main factor related to disengaging from the game, whereas in-app events brought users back to the game. These in-app events and updates could elicit enjoyment and excitement, “It is fun to see new things” and “New Pokémon!!!”

To explore gameplay within the broader context of family, parents were asked to identify 3 goals for themselves or their families. The 2 most cited goals were having fun (114/160, 71.3%) and spending more or quality time with their children (113/160, 70.6%). The third most reported goal was to exercise more (74/160, 46.3%). Making more money was the least reported goal (20/160, 12.5%). Most parents reported that playing Pokémon GO helped them to meet 2 or more of their family goals (107/155, 69.0%), and another 33% of parents reported that gameplay helped them meet 3 or more of their goals. Both parents and children cited multiple reasons for gameplay, yet “spending time with family” was the most cited reason. The majority of dyadic gameplay occurred between a child aged 10 years or younger and a parent (107/160, 66.8%). Parents reported gameplay with sons most often (111/160, 69.4%), and children reported gameplay with mothers most often 87% (27/31). Interestingly, more than half of the parents (107/160, 66.8%) reported playing Pokémon GO *for their child*, even when the child was not physically present. Table 6 provides the social perspectives of gameplay.

### Profiles of Family Health

For aim 2, Pokémon GO was investigated within the context of family health. First, self-reported parental beliefs toward engaging in healthy lifestyle behaviors were relatively high. The average score was 75.00/90.00 (n=160; SD 10.2). Parental indicators of health behavior were obtained from retrospective self-reports of sedentary and PA behaviors. Sedentary behaviors were categorized into television or video watching, video game playing, or surfing the internet or Web. Watching 1 to 3 hours of television daily was the most frequently reported sedentary behavior (n=153, mean 2.71 [SD 1.93]). Surfing the internet accounted for 1 to 2 hours of daily sedentary behavior (n=150, mean 2.43 [SD 1.59]), with fewer parents reporting that they played video games (98/160, 61.3%).

**Table 4:** Facets of Pokémon GO gameplay.

Aspect		Parent self-report (n=160), n (%)	Parent reporting on their child’s gameplay (n=160), n (%)	Child self-report (n=31), n (%)
**Played game while doing something else (eg, walking somewhere and waiting in line)**				
	Yes	145 (90.6)	122 (76.3)	26 (84)
	No	11 (6.9)	34 (21.3)	4 (13)
	Missing	4 (2.5)	4 (2.5)	1 (3)
**Number of times game checked throughout the day**				
	0	6 (3.7)	0 (0.0)	12 (39)
	1	21 (13.1)	73 (45.6)	2 (6)
	2 to 3	51 (31.9)	56 (35.0)	10 (32)
	4+	78 (48.8)	27 (16.9)	7 (23)
	Missing	4 (2.5)	4 (2.5)	—
**In the past 7 days, number of episodes gameplay lasted for more than 30 min**				
	5+	62 (38.8)	28 (17.5)	5 (16)
	3 to 4	47 (29.4)	37 (23.1)	6 (19)
	2 to 3	38 (23.8)	68 (42.5)	17 (55)
	0	9 (5.6)	23(14.4)	3 (10)
	Missing	4 (2.5)	4 (2.5)	—
**Participated in in-app events**				
	Yes	127 (79.4)	58 (36.3)	21 (68)
	No	29 (18.1)	97 (60.6)	10 (6)
	Missing	4 (2.5)	5 (3.1)	—
**Participated in community-sponsored Pokémon GO events**				
	Yes	91 (56.9)	—	10 (32)
	No	65 (40.6)	—	21 (68)
	Missing	4 (2.5)	—	—
**Purchased portable phone battery for gameplay**				
	Yes	96 (60)	—	11 (36)
	No	60 (37.5)	—	20 (65)
	Missing	4 (2.5)	—	—
**Increased phone data plan**				
	Yes	28 (17.5)	—	3 (10)
	No	128 (80.0)	—	28 (90)
	Missing	4 (2.5)	—	—
**In-app purchases**				
	Yes	113 (70.6)	—	—
	No	45 (28.1)	—	—
	Missing	2 (1.3)	—	—

**Table 5:** Engagement with Pokémon GO from parent perspective.

Perspective		n (%)
**Stopped gameplay (permanent or temporary; n=156)**		
	Yes	26 (16.3)
	No	130 (81.3)
**Reason for disengaging from game (permanent or temporary; n=26)**		
	Bored or lost interest	11 (42)
	Too busy	6 (23)
	Addiction	2 (8)
	Weather (unfavorable)	2 (8)
	Completed game or level	2 (8)
	Screen time monitoring	1 (4)
	Game used too much phone memory	1 (4)
	Technical bug	1 (4)
**Reason for re-engaging with game (n=156)**		
	In-app events or updates	30 (57.7)
	Child asked	10 (19.2)
	Fun, interesting	3 (5.7)
	Free time	2 (3.9)
	Friends	2 (3.9)
	Weather (favorable)	2 (3.9)
	Change in work, social circumstance	2 (3.9)
	Child punishment over	1 (1.8)

**Table 6:** Social perspectives of gameplay.

Aspects of gameplay		Parent (n=160), n (%)	Aspects of gameplay	Child (n=31), n (%)
**Typical gameplay (check all that apply)**				
	With a child	97 (60.6)	With a parent	23 (74)
	By myself	80 (50.0)	By myself	4 (13)
	With family or friends	30 (18.8)	With family or friends	4 (13)
**Played game with**				
	Son	111 (69.4)	Mother	27 (87)
	Daughter	59 (36.9)	Sibling	19 (61)
	Niece or nephew	41 (25.6)	Father	17 (55)
	Grandchild	6 (3.8)	Grandparent	7 (23)
	Other	19 (13.1)	Childcare provider	2 (7)
**Enjoyed game because**				
	It helps me spend time with family	122 (76.3)	It helps me spend time with family	24 (77)
	It helps me to go outdoors	113 (70.1)	It helps me to go outdoors	20 (65)
	It makes regular walks interesting	111 (69.4)	It makes regular walks interesting	0 (0)
	I like physical activity	90 (56.3)	I like physical activity	0 (0)
	I am a Pokémon fan	48 (30.0)	I am a Pokémon fan	21 (68)
	I consider myself a gamer	18 (11.3)	I consider myself a gamer	13 (42)

**Table 7:** Retrospective physical activity before and after playing Pokémon GO.

Physical activity type	N	Before playing Pokémon GO, mean (SD)	After playing Pokémon GO, mean (SD)	*P* value
Mild activity (min/day)	—	21.13 (11.72)	26.39 (11.38)	—
Mild activity (min/week)	98	79.59 (68.06)	102.95 (73.50)	.001
Moderate activity (min/day)	—	20.92 (14.11)	25.11 (13.03)	—
Moderate activity (min/week)	131	60.45 (62.82)	82.21 (72.91)	<.001
Strenuous activity (min/day)	—	16.57 (15.49)	18.57 (15.44)	—
Strenuous activity (min/week)	141	48.50 (60.69)	56.00 (66.17)	.03
Leisure score index^a^	160	38.25 (25.84)	48.04 (25.96)	<.001

^a^Leisure score index: frequency of each physical activity level × corresponding metabolic equivalent of task value.

Using the Godin-Shepard Leisure-Time Physical Activity Questionnaire, PA of parents was retrospectively evaluated before and after playing Pokémon GO. To account for variations in exercise routines (eg, Monday-Wednesday-Friday), weekly minutes of PA (vs daily) and an overall LSI were used for pairwise comparisons. Pairwise *t* tests compared PA levels before and after playing Pokémon GO. We applied a Bonferroni correction for the multiple comparisons of PA levels and overall LSI (mild PA, moderate PA, strenuous PA, LSI, *P*=.01). We found playing Pokémon GO significantly increased minutes spent in mild and moderate PA per week and overall LSI (Table 7). Although the LSI was relatively high for this sample before gameplay, LSI significantly improved from 67.5% (108/160) to 80.0% (128/160) after gameplay.

Correlations between family characteristics and health variables (parental healthy lifestyle beliefs, parental PA, and child perceptions of parental influences on PA) were assessed. Parents’ education level significantly correlated with household income (*r*_s_=.395, *P*=.03) and healthy lifestyle beliefs (*r*_s_=.170, *P*=.03). These findings indicate that the more educated the parents, the more likely they were to have higher annual income and beliefs in their ability to engage in healthy lifestyle behaviors. However, analysis of parent-child dyadic data showed that parent healthy lifestyle belief scores (n=30, *r*_s_=.242, *P*=.19) did not correlate with child perceptions of parental influences on PA (n=31, mean 30.06 [SD 5.13]). Instead, child perceptions of parental influences on PA significantly correlated with LSI, both before and after Pokémon GO (n=31, pre-LSI *r*_s_=.503, *P*=.004; post-LSI *r*_s_=.476, *P*=.007). Upon closer examination, the most significant association was found between child perceptions of parental influences on PA *subscale for active parents* and parent-reported minutes per week spent in *strenuous* PA (pre gameplay *r*_s_=.389, *P*=.03; post gameplay *r*_s_=.447, *P*=.02). Parent-reported strenuous activity also significantly correlated with child perceptions of parental influences on PA *guiding support subscale* (pre gameplay *r*_s_=.361, *P*=.05; post gameplay *r*_s_=.378, *P*=.04). These findings suggest that children are more likely to perceive parental influence on PA if the parent regularly engages in strenuous activity and has supportive rules for PA participation.

## Discussion

### Principal Findings

Pokémon GO transcended traditional understanding of digital health and uniquely reached across generations to engage users. Analogous to other research exploring interest in health apps [62,63] and Pokémon GO family research [26], our sample tended to be white, educated, and with higher than average household incomes. Although Pokémon GO sparred many conversations and lessons to be learned [24], it is critical to note that a significant portion of families are underrepresented. From our sample, it is evident that families with young children are co-users of technology. Largely consistent with previous Pokémon GO research [26,30,32-34], our findings show that for stints in time, Pokémon GO promoted physical and social well-being. Findings are presented per the following study aims: (1) to explore the Pokémon GO user experience from a family perspective and (2) to investigate Pokémon GO within the context of family health.

#### User Experience

Findings are presented following facets of the user experience honeycomb (ie, usability, adaptability, accessibility, desirability, and value) [54,64]. Ease of use or *usability* refers to the ease with which users can complete their intended task using a product [61]. For example, Pokémon GO was able to reach a broad audience by designing a free game for use on mobile devices (opposed to requiring users to purchase a specific gaming device). In the United States, approximately three-quarters of all Americans own a smartphone [65]. Similarly, Pokémon GO was and continues to be available across Android and iOS platforms. Yet, as a freemium game (ie, where additional features may be purchased) Pokémon GO consumed up to 50% of mobile phone–based micro transactions shortly after release [66]. We found more than 70% of parents reported in-app purchases. This was an unanticipated finding and follow-up questions regarding specific purchases were not part of the initial survey. However, our findings suggest that usability in children or family units may differ than that in individual or adult populations. We found that parents leveraged gameplay to punish or reward child behavior. For instance, parents reported, “We will use a Pokémon excursion to the zoo or a park to reward good behavior” or “Planned day outings...based on where the nest sites are.” Similarly, a child’s usability may be influenced by parental monitoring as young children are less likely to have their own mobile phone or have freedoms related to in-app purchases. In other Pokémon GO family research, parents believed that gameplay was associated with danger and threatened safety [26,48]. Thus, in families, individual gameplay particularly for young children may be linked to interpersonal relationships and influence usability.

Elements of emotional design (behavioral, reflective, and visceral) [67] that invoke emotional reaction to material objects should be considered part of the user experience [54]. *Desirability* refers to the power of a brand or image and is dependent on a user’s context [54,61]. Through collectable cards, games, books, and animated television series launched in 1996 in Japan, Pokémon remains one of the world’s most popular entities [68]. Yet, in our sample, over 40% of parents (69/160, 43.1%) never engaged with the Pokémon brand before Pokémon GO. Conversely, children identified with the Pokémon brand through television shows (28/31, 90%) and card collecting (23/31, 74%). In our sample, gameplay was most often between a child aged 10 years or younger and a parent. During middle childhood, children’s connections with their parents and family are of tremendous importance for their social and emotional well-being [69]. Although not longitudinally collected, parents’ responses to initial uptake of Pokémon GO signaled positive emotion associated with gameplay, “I love the smile my daughter gets when she catches a Pokémon,” “It is a great way to spend time with kids and their friends,” and “It is exciting to walk together and share.” Research shows that parents more directly influence learning when they choose to engage in activities with their children [23], and play, even digital play, helps families learn and connect [70,71]. Six conditions that lead to productive JME include mutual engagement; dialogic inquiry (inspire collaboration with others); cocreation (use media to build things); boundary crossing (span time and setting); intention to develop (at least one partner aims to grow through the activity); and focus on content not control (jockeying for control are kept to a minimum) [23]. In addition to these 6 conditions, Sobel et al [26] found Pokémon GO had other qualities that encouraged productive JME. Pokémon GO hinged on going outdoors, walking, and working in teams; gameplay relied on dynamic outdoor context; gameplay facilitated social connections outside of the family; and gameplay could be shared differently depending on how parents want to participate [26].

Explosive contagion *going viral* occurs when the transmission of a phenomenon becomes strong enough to overcome reluctance [72]. The spread of social phenomena mimics situations in which the willingness of individuals to adopt something new depends not only on the intrinsic value but also on whether acquaintances will adopt this product or not [72]. Findings from adult literature showed that when participants perceived strong social pressure from people around them, they were likely to play Pokémon GO while walking [35]. Individuals are embedded in social contexts; therefore, interpersonal and social processes are recognized as powerful levers for behavior change [73]. Likewise, a product is likely to be adopted if it is *accessible*. Although Pokémon GO was free to download, research has suggested that minority populations faced greater challenges playing Pokémon GO due to incentivized gameplay toward advantaged areas and away from rural places and places with larger minority populations [74,75]. Thus, ethical design must develop at an early stage and consider digital divide, equity, privacy, and autonomy [76].

Aligning product features with user needs drives a product’s *value*. Foremost, our findings reinforce previous evidence [26,34,48] that Pokémon GO provided an opportunity to support family values and fostered family bonding. The challenge of balancing work, life, and parenting responsibilities creates a uniquely stressed situation for parents [77-79]. Time, as a commodity, is extremely valuable to parents [80]. Recognizing the interaction between personal characteristics and situational factors, identity-based motivation (IBM) bridges psychological and social literature to facilitate integration with goal theories [81]. IBM assumes that identities are dynamically constructed in context and people are more likely to take action if something is identity-congruent [81,82]. The challenge of leveraging identity is that the same attribute can be motivating or demotivating within context, depending on the meaning and interpretation of difficulty [83]. Decisions and behaviors are often the result of goals and motives people possess [84]. Activating a goal can influence many aspects of behavior, including how people perceive, evaluate, and feel about the world around them [84]. The number of means attached to a given goal vary, and conversely, so may the number of goals attached to a given mean [85]. For example, in our sample, playing Pokémon GO served multiple goals through a single mean. Pokémon GO served the 3 most cited parent goals: have fun, spend more or quality time with their children, and get more exercise. Multifinality set denotes the number of goals linked to a mean, which may affect the perceived value of the mean or *bang for the buck* [85]. For a period of time, Pokémon GO was an extremely efficient use of time for families. However, goal systems are highly variable and context-dependent [85]. A different set of goals to the same mean may change in different context or circumstances [85]. Differently resourced families have different time, energy, and budget available for supporting media in a media-rich world competing for our attention [18,23]. Pokémon GO was able to re-engage nearly 60% of users who stopped the game using novelty via in-app events and updates. In our study, as context and circumstances changed over time (eg, weather and work schedules), gameplay became less *desirable* to some families. When specifically asked about re-engagement, for some, open-ended comments swayed negative, “My obsessed son,” “My kid’s bugging me about it,” and “I don’t always want to be on my phone or my son to be on my phone.” This highlights heterogeneity not only between families but also within families that can occur over time. Therefore, opposed to sustained engagement, research using digital technologies to support health should focus on effective engagement, sufficient to achieve intended outcomes [86], particularly in family interventions.

#### Family Health

Our findings support previous research [30,36,38], showing that when played as designed, Pokémon GO was associated with increases in PA. We found significant improvements in mild and moderate PA after Pokémon GO uptake, with the largest gains observed in mild activity (approximately 23.36 min/week). In addition, 20 participants (20/160, 12.5%) shifted from being classified as *insufficiently active* to *active* after playing Pokémon GO. We believe that part of Pokémon GO’s success as a health app was the stealth health approach accomplished through gameplay. Stealth health is an intervention approach where the target is a side effect but not the primary motivator of participation (also a common gamification technique) [87]. Using this approach, behavior change occurs by shaping existing situations (eg, relaxing after dinner by watching television) into preferred situations (eg, relaxing after dinner by playing a location-based game).

Research conducted in college students show that variables from the theory of planned behavior (attitude, subjective norms, and perceived behavioral control) were significant predictors of behavioral intentions for gameplay [33,35]. Behavior change techniques (BCTs) are theoretically derived behavioral determinants identified for a given target [9]. There is empirical support favoring interventions that incorporate BCTs to increase self-efficacy and PA versus comparator [10,12,88,89]. As such, we found the following BCTs used in Pokémon GO: feedback and monitoring, reward and threat, goals and planning, shaping knowledge, and social support. The same BCTs found in Pokémon GO are also common to both gamification techniques [90-93] and pediatric health promotion apps [87,94,95]. Across BCT literature [10-12,88,89], planning is commonly used to increase self-efficacy, and self-monitoring is commonly used to increase PA. Although self-efficacy has been associated with PA behavior change [12,96], the influence of parental self-efficacy on PA evidence of a child is inconclusive [60,97]. In our study, parental beliefs toward healthy lifestyles did not correlate with child perceptions of parental support for PA. Rather, we found that parental behaviors, particularly strenuous PA, associated with child perceptions of parental support for PA. In a systematic review of mobile apps, role modeling was the only predictor of PA in children aged 6 to 13 years [95]. Other research demonstrates that perceived parental norms and role modeling are associated with healthy lifestyle behaviors in school-aged children [2,98,99], particularly influencing time spent outdoors [99].

### Limitations and Future Research

Limitations of this study should be noted. The study design and convenience sample used weaken the strength of the study. We were unable to determine comparisons by child age (eg, <10 years vs ≥10 years) or gender because of limited distributions. Survey development and use of the game were conducted by 1 individual on the project; a diverse set of inputs would strengthen future research. Findings presented here reflect the lag time between academia and health relative to the pace of technology. The convenience sample data were collected 6 months after the initial release of Pokémon GO and skewed toward educated white populations. Research is needed to advance the real-world implementation of digital health interventions [22,100]. Specifically, research is needed to understand how all different types of families use media together, use competing technologies, and create equitable opportunities for JME in resource-limited families [23,101]. JME presented here is within the context of parent-child. Yet, JME is a much broader concept to include digital learning across individuals, including peer, sibling, and family. We briefly explored these relationships, discovering that 61% (19/31) of children reported playing Pokémon GO with a sibling. Finally, data were collected through self-report, and changes in PA were retrospective self-report, which may introduce recall bias and social desirability.

### Conclusions

We believe this research extends the science by highlighting the Pokémon GO user experience and within the context of family health. Our findings parallel other research [102-104] suggesting that popular *pop* culture may be leveraged to promote health. However, gender (or gender neutral) associations may exist with different brands. As technology becomes more ubiquitous, equity concerns persist for reasons that transcend mere access to these tools [23,101]. Recognizing families as co-users of technology, particularly in families with children aged 5 to 10 years, digitally supported behavior change interventions that incorporate JME strategies should reflect the child’s developmental stage and the dynamic nature of media use within the family. Within the ever-changing context of family life, we found a balance between the stability of family connectedness and everyday life. Our findings further suggest that Pokémon GO’s success with children and families may be attributed to how gameplay became entwined with and strengthened important family values, while stealthily serving multiple other goals. In busy families, the efficiency of Pokémon GO justified gameplay. Although parents are traditionally recognized as agents of change for family health [2], findings from this study suggest that children aged 10 years or younger may serve as reciprocal agents of change by promoting engagement with digital health interventions. Pokémon GO underscored the potential for digital health, demonstrating that a single app can touch the lives of millions. With the anticipated release of Niantic’s Harry Potter AR game in 2018 [105], another opportunity to broaden the science in children and families appears on the horizon.

## Abbreviations

BCT: behavior change technique
IBM: identity-based motivation
JME: joint media engagement
LSI: Leisure Score Index
PA: physical activity
